# Foreign Summary

**Published:** 1840-03-28

**Authors:** 


### FOREIGN SUMMARY.

*Clinical Lecture on Fractures, delivered at University College Hospital.* By Samuel Cooper, Esq.—Gentlemen, as nearly one-third of the patients under my care in this hospital have consisted of persons, with broken bones, I avail myself of so favourable an opportunity to notice some of the most interesting points about this numerous group of cases, the observation of which has been amply within your reach. Although University College Hospital does not contain a large number of patients—not more than 130, on account of the present state of its funds—the great number of accidents brought to it every year, and the many operations annually performed in it, are too well known to the profession and public at large to require any observations from me.

*Fracture of the Tibia and Fibula—Retarded Union.*

Charles Arnott, aged 39, admitted July 7, 1839. The accident occurred from a fall, when the man was in a state of intoxication. The fracture of the tibia was exceedingly oblique; the angular displacement was considerable, and the sharp end of the tibia projected against the skin, a little above the ankle. The limb was put up on M'Intyre's apparatus, and a compress applied so as to keep the bandage from pressing on the pointed extremity of the tibia.

No union having taken place, on the 7th of October the limb was first carefully bandaged, two lateral pieces of strong pasteboard applied, and then another roller, wetted with a solution of starch. The latter having dried, formed a stiff support for the broken limb, or what has been called by the French an *irremoveable apparatus*. Under the protection of this, the patient was directed to get up, and to try to walk about the ward with crutches.

On the 22d of this month the apparatus was removed, and the state of the fracture examined. The fibula is united, and the tibia is evidently uniting also: so that, in another week or two, the cure will be sufficiently advanced to enable the man to discontinue all mechanical support of the limb.

This case, besides exemplifying the usefulness of the starch-stiffened roller, and of lateral pressure on a very oblique fracture that is slow in uniting, illustrates the occasional efficiency of letting the patient get up and walk about, so as to excite more action, as it were, in the process of union, according to the maxim long ago suggested by the celebrated John Hunter.

*Thigh-bone broken a second time—Fracture of the Provisional Callus.*

Gentlemen, I presume that you know the interesting fact, that when a broken bone unites, an external or temporary callus is formed in the first instance around the outside of the fracture, constituting an excellent contrivance, whereby the ends of the fracture are supported, connected together, and kept steady until nature has had time to produce the permanent callus, by which the ends of the bone are to be firmly and inseparably united directly to one another. While this forms precisely between the surfaces of the fracture, the temporary or provisional callus is like a broad hoop, as it were, placed around the broken part. After having served its transient, but very important office, it is taken away, and then the strength of the bone depends entirely on the permanent or definitive callus. At the period when a surgeon usually discontinues the splints, or other apparatus, and the fracture is considered to be united, the formation of the permanent callus is scarcely begun, and the degree of firmness in the fractured part depends entirely on the temporary callus, which is never half so strong as that of later production. Hence, if the limb be exerted too violently, or too much weight be imposed upon it, or any fall take place, the temporary callus is liable to yield or break.

The case of John Kelly, aged 24, exemplifies what I have now been explaining. He was admitted into this hospital on June 8th with an oblique fracture of the left thigh, about the middle of it, and also a fracture of both bones of the right leg. On the 16th of August, or at the end of about nine weeks, he was discharged cured, both the thigh and leg appearing to be firmly united, and the limbs symmetrical and of the same length.

On the 19th of August he was re-admitted, in consequence of a fall against the edge of his bed, and a fracture of the thigh-bone in the same situation as before. This second fracture was put up in the straight position, and with the long splint.

*Sept.* 20.—On examination of the thigh, the bone was found to be uniting again.

*Oct.* 16.—Union firm again—limb of the same length as the other—long splint discontinued, and merely pasteboard and a roller applied.

*Oct.* 22.—All mechanical support removed, and the man allowed to use his crutches again.

This case, gentlemen, shows you several interesting points.

1. The liability of the provisional callus to be fractured, if exposed to external violence, or undue efforts, or too much weight upon it, previously to the completion of the definitive callus.

2. The truth of Dupuytren's calculation, that when the provisional callus breaks, about as much time is requisite for the second cure as for the first.

3. The case gives you a fine example of an abundant provisional callus, which is felt to be of great thickness, but which, if the man come under our notice again a year hence, when the definitive callus will be complete, will all have been removed, and the bone reduced to its natural size.

4. Convenience of the treatment with the patient lying on his back.

*Compound Fracture; Wound healed without Inflammation, or any serious Constitutional Disturbance; Union as prompt as that of a Simple Fracture.*

These circumstances are exemplified in the case of Sarah Bishop, admitted under me, Sept. 10, 1839.

The limb was placed on M'Intyre's apparatus—the bones adjusted, and lint dipped in cold water laid over the wound.

14th.—Wound had completely healed, but a trivial slough afterwards separated, which left a small sore, that was perfectly healed by 5th of October.

*Fracture of the Frontal Bone communicating with the Ethmoid Cells, or the Frontal Sinus, and attended with Emphysema, and Concussion of the Brain.*

Traumatic emphysema of the eyelids is not very uncommon. Dupuytren relates a case where it was presumed to have arisen from a fracture of the os planum of the ethmoid bone, or else of the lachrymal bone. He gives the particulars of another case, in which traumatic emphysema proceeded from a laceration of the pituitary membrane; opposite the junction of the lateral nasal cartilage, which was itself separated from the bones of the nose. In a third instance, recorded by the same illustrious surgeon, the emphysema was occasioned by a fracture of the frontal sinus. I will now enter into some explanation of the case which was lately brought to this hospital, and which will be found to present several points of interest:—

Arthur Hallam, aged 36, admitted June 22, 1839, footman to His Royal Highness the Duke of Sussex; of temperate habits, and good constitution.

About half-past 11, A. M., as he was proceeding from the terminus at Euston-square, in the last carriage of the railway train an engine came up behind at full speed, and struck the carriage with immense force, whereby he was thrown forwards with great violence, and he received a severe blow above the right eye which completely stunned him. In this state he was brought to the hospital: pulse small and feeble, pupils of the eye dilated. Notwithstanding the ecchymosis and swelling, a fracture of the superciliary ridge was perceptible, accompanied by emphysema, and a crepitation of air in the cellular tissue around the orbit. There was hemorrhage from the nose and mouth.

A few minutes after his admission he became sensible, and his pulse began to rise. He now complained of severe pain in the head.

At one o'clock I saw him. As he had then rallied considerably, and the pulse was fuller and stronger, ℥xvj. of blood were taken from the arm; all the fore part of his head was shaved, and covered with linen wetted with cold water, and gr. v. of the chloride of mercury were prescribed, and followed by the senna mixture every four hours.

Ten o'clock, P. M. Severe headach yet persists; *double vision;* pulse being full and hard ℥xvj. more blood were drawn, and the evacuation afforded much relief.

*June* 23. Headach still severe; *pain in the epigastrium, dimness of vision,* and *intolerance of light.* Has had *shiverings.* Bowels have been freely opened.

Eighteen leeches applied to the epigastrium, cold lotion continued, and two table spoonsful of the saline antimonial mixture directed to be taken every four hours. The bleeding from the leeches gave considerable relief.

*June* 24. General improvement, emphysema lessened, pulse reduced to 85.

*June* 25, nine, A. M. Further amendment; intolerance of light and dimness of sight removed. Cold lotion and saline aperient mixture continued.

Ten, P. M. Feel of coldness in the course of the spine; more headach; *vision indistinct again.* Twenty leeches applied to temples, followed by fomentations.

*June* 26. Symptoms relieved, emphysema more diminished, distinct vision restored, pulse soft and regular, little appetite. Cold lotion and saline aperient mixture continued.

*June* 29. Severe pain in the right temple having come on again, fifteen leeches were applied, and gave great relief. The bowels being too open, the sulphate of magnesia omitted in the mixture.

*July* 4. Allowed to get up and take a little beef tea, for the first time. *Light yet inconvenient* to the patient when he looks at a window. Cold lotion and mist. ant. tart. continued.

*July* 20. Discharged, cured. The patient called at the hospital a few days after this date, complaining of vertigo and headach, for which he was cupped, and restricted to a very low diet. This plan at length restored him to perfect health.

This case, gentlemen, gives you an instance of the following circumstances:—

1. Fracture of the frontal bone, extending either to the frontal sinus, or os planum, with traumatic emphysema.

2. Fracture with concussion of the brain.

3. *Dilatation of pupils,* frequently noticed in the early stage of concussion.

4. Concussion succeeded by *diplopia* and indistinct vision, of which we have lately had other examples in the hospital.

5. The long duration of headach, intolerance of light, and other symptoms after concussion of the brain, and their great propensity to return.

6. The signal efficacy, in such cases, of bleeding, leeches, cold lotions, calomel, and antimonial saline medicines, with a rigorously low diet.

7. The quick appearance of emphysema after a fracture of the foregoing kind; its limited extent, so different from what sometimes follows an injury of the lungs themselves, and the usual commencement of its subsidence in three or four days after the accident.

---

*On the employment of a new vegetable, Monesia, in Medicine.* By Dr. G. J. MARTIN ST. ANGE.—A vegetable substance, called *monesia,* has lately been imported from South America, in the form of hard thick cakes, weighing about five hundred grammes, (9215 grains.) These loaves, which are flattened, and have paper, of a yellow colour, adhering to them, are composed of the extract, prepared in the country, from the bark of a tree, whose botanical name is not known. M. Bernard Derosne, the druggist who introduced it, informs me that some travellers call the monesia bark *goharem,* and others, *buranhem.* But what is of more importance, is, that the naturalists who have examinedit, think that the tree which furnishes it, is a chrysophyllum.

The extract is of a deep brown, and very friable; when broken, it looks like a well roasted cocoa-nut. It is entirely soluble in water; and its taste, which is at first, sugary, like liquorice, soon becomes astringent, and leaves behind a well marked and lasting acid taste, which is particularly felt in the tonsils.

The bark of the monesia is smooth and grayish, like that of the plane tree, with this difference, however, that it is much thicker; that its fracture is imbricated, and that its sweet taste forms a strong contrast with the bitterness of the thin laminæ which are detached from the plane.

The chemical analysis of the bark of the monesia, and of the imported extract, according to MM. Bernard Derosne and O'Henry, has demonstrated the presence of the following soluble principles:—1, Chlorophylle; 2, vegetable wax; 3, a fatty and crystallizable matter; 4, glycyrrhizine; 5, an acrid and somewhat bitter substance; 6, a little tannin; 7, an unexamined organic acid; 8, a red colouring matter, resembling that of cinchona; 9, phosphates of lime, with organic acids.

The pharmaceutical preparations which have been made with this substance are—1, an aqueous extract; 2, a syrup, containing thirty centigrammes, (5½ grains) in the ounce; 3, a hydro-alcoholic tincture, containing two grammes (37 grains) per ounce; 4, chocolate, containing thirty centigrammes (5½ grains) in each cake, weighing three decagrammes (7 drachms, 49 grains;) 5, an ointment, containing an eighth part of its weight of extract; 6, monesine being the acrid substance mentioned in the analysis.

The extract contains about eight per cent. of glycyrrhizine, and twenty per cent. of acrid matter.

The following accounts of monesia are already in existence:—1. A manuscript memoir, which is in the hands of the commissioners appointed by the Academy of Medicine. 2. A synoptical table, giving the analysis, some pharmaceutical preparations, and the medicinal preparations of monesia. 3. A very minute summary of these two papers, entitled, "Account of Monesia." 4. An article inserted in the *Bulletin Thérapeutique*.

I will now give a succinct account of the facts which have been published, before mentioning the results which I have obtained, myself.

The medical cases in the synoptic table have been drawn up by several physicians in Paris; they give the nature of the disease, the sex, the profession, the age, and the constitution of the patient; the mode of treatment, and duration of the disease, the termination; and, lastly, the remarks suggested by each method of treatment.

M. Alquié, professor of internal pathology at the Val-de-Grâce, found—

1. That of forty-two soldiers attacked with diarrhœa, of different degrees of severity; thirty-six were cured in twelve days; twenty-four by the extract of monesia, given in pills, in the dose of from eighty centigrammes, to a gramme (14½ to 18½ grains) a day; and twelve by the tincture, administered as a clyster, in the dose of eight grammes (147½ grains) in two hundred and fifty grammes (4607½ grains) of bran water.

2. That in two cases of menorrhagia, the extract and the tincture of monesia given internally, soon calmed the pain, and stopped the uterine discharge.

3. That in four women attacked with profuse leucorrhœa, the extract of monesia given internally, and the diluted tincture injected into the vagina, were beneficial.

4. That in two cases of hæmoptysis, where bleeding, ligature of the limbs, and ordinary astringents, had been employed without advantage, the extract of monesia succeeded completely; and that several chronic cases of bronchorrhœa were benefited by the syrup of monesia, which was sometimes combined with opium.

M. Baron cites—1. A very remarkable case of chronic inflammation of the vagina, of a syphilitic kind. No advantage had attended the previous use of baths, local bleedings, emollient and astringent injections, the nitrate of silver; a year later, the diluted supernitrate of mercury, sulphureous baths, leeches, and the repeated application of blisters and sinapisms, were equally useless. In spite of these remedies, the discharge from the vagina became more abundant. Injections were then used containing thirty grammes (552 grains and 9-10ths) of the extract of monesia in a hundred and fifty grammes (2764½ grains) of water. In eight days the discharge was much diminished, and in three weeks the patient was cured. The discharge returned in a month, but again yielded to the same injections.

2. A case of leucorrhœa. The discharge was copious, of a yellowish white colour, and accompanied with pains in the groins and lumbar regions; baths, leeches, and injections of mallow water and laudanum, had produced no benefit. Injections of monesia, in the proportion of thirty grammes (552 grains and 9-11ths) to a hundred grammes (3317½ grains) of water, were employed once a day, and the patient was cured in a fortnight.

3. Several cases of diarrhœa, which resisted the means generally used, were cured by the extract of monesia given internally, and clysters containing the tincture, in different proportions.

M. Buchez has employed the extract of monesia, and has remarked, that it delayed the progress of caries in the teeth, and that when combined with opium, it often soothed the pain more effectually than the opium alone. He recommends the employment of the tincture to keep the gums in a healthy state.

M. Daynac speaks of the good effects he has obtained from the preparations of monesia

(the syrup, lozenges, and paste) in several cases of the chronic catarrh of the old, in dyspeptic persons, and in the third stage of phthisis. He also cites remarkable cases of scrofulous engorgement, much benefited by the use of the tincture of monesia, in the dose of eight grammes (147½ grains) daily, continued for a greater or less time. Lastly, the extract of monesia in pills, in the dose of sixty to ninety centigrammes (11 to 16½ grains,) has been very serviceable in uterine discharges.

M. Laurand speaks of a well marked case of scurvy which he cured with monesia. The patient had had frequent epistaxis, which had several times required the nostrils to be plugged. He was made to inspire acidulated water by the nostrils, containing thirty grammes (552 grains and 9-10ths) of the tincture to a pound of water. This stopped the hæmorrhage; but when the same thing had been done with acidulated water not containing monesia, it had not succeeded. The patient also took from a gramme to a gramme and a half (18½ to 27¾ grains) internally, every day. The same physician has ascertained the efficacy of monesia in a great variety of circumstances, particularly in gangrenous eschars on the sacrum.

M. Manec has employed the different preparations of monesia with success:—

1. In a man who, for six years, had had a large herpiginous ulcer in the bend of the groin, which had resisted every kind of treatment, and which rapidly improved under the use of monesia ointment.

2. In a great number of aged women, labouring under diarrhœa, and in persons affected with chronic bronchitis.

M. Monod has furnished some very interesting cases; some, of ulcers of the nose, and others of affections of the intestinal canal.—The ulcers were dressed with the powdered extract, and cured in a few days. In the other cases, the extract given in pills to the amount of sixty to a hundred and twenty centigrammes (11 to 22 grains) daily, was perfectly successful.

M. Payen, who has employed monesia in a great number of cases, has seen a patient in whom leucorrhœa was considerably increased by this medicine, administered two different times; the monesia was then tried as an injection, and the discharge, which had hitherto resisted every remedy, disappeared, and did not return. The same practitioner cites two cases of uterine hæmorrhage, where the patients were obliged to keep their bed for a fortnight at each menstrual period, and in which the monesia brought back the discharge to its healthy standard. Lastly, M. Payen has succeeded in cicatrizing an ulcer in the lower jaw, which for ten months had resisted every kind of treatment, both internal and external; and in healing ulcerated chilblains, by means of the ointment and the powdered extract of monesia.

Thus we see that monesia has been employed both externally and internally. It has been frequently administered during the chronic stage of bronchitis, usually alone, but sometimes combined with opium, and in the greatest number of cases it has seemed to act advantageously upon the disease, the expectoration and respiration being rendered more easy.

In many cases where pulmonary hæmorrhage was prolonged, having resisted various and generally efficacious remedies, the extract of monesia has stopped the spitting of blood.

In weakness of the stomach, monesia has a very favourable influence on digestion, and secondarily on nutrition. This medicine has also been very beneficial in chronic enteritis; it has chiefly succeeded against diarrhœa, from whatever cause it arose.

The efficacy of monesia taken internally has been less marked in leucorrhœa than in diarrhœa; yet it has been useful in the majority of patients who have taken it; but injections have been more advantageous.

In every case of uterine hæmorrhage where monesia has been given, it has succeeded in moderating and suppressing the discharge more readily than the other remedies which had been previously used.

Monesia has also been of great advantage in scorbutic and scrofulous affections, and has always benefited ulcers of a bad character, whether the ointment, or the pure extract powdered, or the acrid substance contained in it, has been employed.

Such is the compendium of the cases hitherto published, with the exception of four by M. Forget, which are the basis of the article that he has published in the *Bulletin Thérapeutique*, and which, as he says himself, neither tell for nor against monesia.

We may say, therefore, generally, that monesia shows its maximum of power in diseases of the digestive organs, in hæmoptysis, uterine hæmorrhage, and ulcers of the skin, or of the mucous membranes, at their origin. A remarkable point in this remedy is, that although it is gifted with energetic powers, and has acted upon the tonsils or upon ulcerations as an active stimulant, it has never irritated the stomach as tonics, properly so called, often do. In order to form a due estimate of its relative activity, we must not forget that it has always been employed after the exhibition of other remedies.

I now come to my own cases, the general results of which may be stated as follows:—

Monesia, when exhibited internally, in the dose of from 75 to 125 centigrammes (14 to 23 grains) of the extract daily, for eight or ten days, whether in the form of pill, tincture, or syrup, has an immediate effect upon the digestive passages, and quickens the action of the stomach in a very remarkable manner. If the dose of the remedy is pushed to four grammes (74 grains) of the extract, daily, for fifteen or twenty days, the appetite increases, but the patients sometimes experience a feeling of heat in the epigastrium:* tenesmus and obstinate constipation may also come on; hence its action upon the digestive tube should be moderated by diminishing the dose according to the effect produced, and administering emollient or laxative clysters, as may be required.

Monesia ointment may be employed externally upon sores, in every case, but with more or less success, according to circumstances: thus I have seen it succeed in large and excessively painful ulcers, arising from the action of blisters, in sores produced by burns, in varicose ulcers and old wounds; in a word, whenever the sore is painful, and depends on a merely local affection. When this is not the case, and the ulcer is kept up by syphilis, scrofula, scurvy, or cancer, it is impossible to effect a permanent cure by merely applying the monesia ointment, washing the sores with the tincture, or sprinkling them with the extract or acrid principle contained in it. Yet, by employing these different preparations in a proper manner, we may hope to modify the sores, and even to cure them for a time. Generally speaking, the ointment, when applied to a sore, calms the local pain; the tincture thus used, produces a sensation of heat, which ceases immediately; the powdered extract more or less excites the sore, and the acrid principle in powder, when well prepared, has a special activity greater than caustic: hence it is a powerful remedy against fungous or atonic ulcers of a bad appearance; but as soon as these sores become painful, and especially when they are covered with a whitish pellicle, the use of the acrid principle should be discontinued; for it is usually this pellicle which, by preserving the surface of the sore from contact with the air, and perhaps by becoming partly organized, produces cicatrization.

I have said expressly, that it is impossible to obtain a lasting cure of syphilitic or cancerous sores by the mere external use of this remedy; in such cases, therefore, we must have recourse to a specific treatment capable of acting on the system. I have found, that in order to effect the cure of scrofulous ulcers, the monesia must be employed internally, for five-and-twenty or forty days, and even longer, according to the case; and this in large doses, such as four of five grammes (74 or 92 grains) of the extract daily, in the form of pill, tincture, or syrup. In this way I have succeeded in curing or benefiting several scrofulous patients. Here follow two remarkable examples:—

*Case* I.—A young man of 17, a printer, born of very healthy parents, came to see me in February, 1839, to have the little finger of his left hand amputated. On looking at the diseased parts, I saw it was a scrofulous affection of only eight months' standing. The first phalanx was much swelled, the soft parts covering it, were livid, and there were three fistulous openings in the skin; two corresponding to the dorsal part of the phalanx, and the third to its palmar surface. They were surrounded with callous vegetations of a brownish colour, and communicated with one another by means of subcutaneous fistulous passages. By introducing a blunt probe into the sores, it was easy to reach the bone of the finger, and to ascertain the detachment of the skin and the caries of a portion of the phalanx. The suppuration was serous, yellowish, of a faint odour, and contained some flakes of a substance which seemed carious. Strong pressure of the diseased tissues occasioned hardly any pain. On the back of the hand and left elbow, there was also a swelling of the skin and of the subjacent parts, looking like the little finger. The swelling and livid patch extended from the elbow* to the inside of the bend of the arm; its centre was ulcerated, and covered with a thick crust, which, according to the patient's report, was renewed every two or three days.

I began by sprinkling the acrid principle of monesia on the small sores of the finger. After some days' dressing, the swelling of the soft parts began to diminish, and at the end of about twenty days, the fistulous openings entirely closed. The diseased tissues at the back of the hand then ulcerated, and the acrid principle being employed as above mentioned, in a few days a cure was effected. There remained only the sore upon the elbow, which had been purposely dressed with cerate. It continued to suppurate, and to be covered from time to time with a fresh crust.

The patient was in this state when I presented him to Dr. Bailly, who had been commissioned by the Academy to report on the effects of monesia. The affection appeared to him to be evidently scrofulous, and the result obtained, to be very satisfactory. The disease, however, soon reappeared; the fistula of the finger began to suppurate again; there was swelling and livid redness of the soft parts, with engorgement and induration of the back of the hand; the sore on the elbow became larger and deeper. The patient now entered the hospital of St. Louis, where he had internal medicines as well as fumigations, sulphurous baths, &c. In a month, he came out, with the diseased parts in a worse state than ever. I now prescribed the internal use of monesia—namely, twelve pills, each containing 20 centigrammes ($3\frac{1}{2}$ grains,) and two

* Showing that it *does* irritate the stomach, contrary to the assertion made a few lines before.—*Translator.*

* The original here has *cou*, but this must be a misprint for *coude.*—*Translator.*

spoonfuls of the tincture. The sores were dressed with common cerate. Under this treatment, the patient was cured in thirty-five days. Nevertheless he continued to take five pills a-day, till the fiftieth day.

Since July, the diseased parts have been constantly improving, and a lasting cure may be hoped for. It is right to state, that in this case the preparations of monesia did not cause tenesmus or constipation, although the patient did not employ any purgative; the only thing he complained of, was too much appetite.

*Case* 2. M. ——, æt. 40, who had always enjoyed perfect health, came to France two years ago, and perceived, in the month of April, 1839, that he had an indolent tumour in the left inguinal region. Several physicians of the capital were consulted, and they ascertained that it was a swelling of one of the superficial lymphatic glands, situated in the bend of the groin. On the 21st of the same month, I was also consulted by the patient. The diagnosis was not difficult, but the point was to know how the tumour would turn out. My prognosis was favourable, like that of all the other physicians, excepting M. Lisfranc, who thought that the swelling of the gland, though slight, depended on a general affection. On the 2d of May the groin continued to swell, and from that time all the other glands of that part, as well as of the left iliac fossa, swelled considerably; and this was soon the case with those of the opposite side. Twenty pages would scarcely suffice to tell all that was prescribed by the physicians, and patiently submitted to by M. ——. No remedy was of any use, except for a short time; and I therefore proposed monesia, in the dose of one hundred and fifty centigrammes (twenty-eight grains) of the extract a-day. The patient at this time was extremely weak, ate but little, and was feverish every day. In a week, digestion had improved; there was a sensible increase of strength, and no fever. The sores were dressed with the monesia ointment. In consequence of these results, I tried to augment the dose of the medicine, and, besides the extract, the patient took two spoonfuls of the tincture, and from four to six of syrup in an infusion of hops. As to the sores, which obviously grew better, the same dressing was continued morning and evening, and every thing promised a speedy cure, when constipation and a most painful tenesmus came on, which obliged us to suspend the treatment. In a few days the sores became larger and larger, fungous and of a bad appearance.

The dressing was then changed—extract of monesia in powder and the tincture being employed; but these remedies were almost as useless as a host of others which were successively tried. It then seemed clear to me that the internal use of monesia had alone produced the improvement, and its use was accordingly resumed, taking care to make laxatives a part of the treatment. For this purpose the patient had two glasses of Enghien water every morning, and an emollient clyster. In a fortnight, the good effects of the monesia were again perceived; and this was more to be attributed to its internal use, as the dressing had been performed with simple cerate.

At present, the swelled glands of the groin are softening and disappearing, without any suppuration. Those of the iliac fossa are diminishing in size; the sores have cicatrized, and the disease, far from attacking the lymphatic glands of the other parts of the body, as is commonly the case, is localized, and is much lessened. The patient eats with a good appetite, sleeps well, and takes exercise three hours a day, which makes us hope for a fortunate termination of the disease.

Another result which I have obtained from the use of monesia, and which has been observed by other practitioners likewise, is its action upon the uterus in cases of meuorrhagia. I will give two instances:—

*Case* 3.—Madame ——, of a plethoric constitution, was attacked, after the catamenial period, with a flooding, which obliged her to keep her bed and seek for advice. After having employed cold drinks, ligatures on the limbs, cupping-glasses, and other revulsives, without success, I made the patient take five monesia pills, each containing twenty centigrammes (three grains and three-fifths.) The next morning she was very weak; the skin burning, the pulse scarcely perceptible, the face pale, and the eyes sunken. She had shivering fits from time to time, a sensation of weight in the loins, transient colic pains, and headach, with sleepiness; and what was more, the hæmorrhage did not diminish. I then prescribed twelve pills of extract of monesia to be taken every hour. The discharge stopped the same day and never returned.

*Case* 4.—Madame ——, aged 20, who had been married six months, had frequent pains in the loins; and in a few days a flooding came on, which obliged her to keep her bed. The hæmorrhage increased, as soon as the patient got up; there was no pain in the abdomen, and no constipation; the pulse was weak and irregular, and from seventy-six to eighty in a minute. Revulsives, cold and acidulated drinks, clysters of cold water, and compresses dipped in iced water and applied to the thighs, had no effect. The ergot of rye was then employed, but as this excited vomiting, it was discontinued, and pills of the extract of monesia were ordered to be taken every hour, until an effect was produced. After fourteen pills the hæmorrhage ceased. The patient then took cold broth at intervals, and in spite of the lightness of this food, the discharge returned in the evening with violence, and again ceased after the exhibition of ten monesia pills.

On the following day, the dose of the medicine was diminished to seventy-five centigrammes (fourteen grains) and in six days the patient was quite well.

Quite lately, I employed the acrid principle in powder, in the dose of fifteen centigrammes (two grains and seven-tenths,) taken in a prune; it was to stop a uterine hæmorrhage, which had suddenly come on during the night; the discharge ceased the same day. But as this case stands alone, additional facts are necessary to prove the power of the acrid principle under such circumstances. In every case, monesia acts in a remarkable manner upon the uterus, when it is not in its natural state. This new medicine may be used in different ways, and it acts on different organs, particularly when they require to be strengthened without too much excitement.

This is confirmed by the following passage from M. Buchez:—

"I have tried the extract of monesia," says this skilful practitioner, "in different affections of the mouth, particularly in inflammation of the gums, and uniformly with advantage. Its application produced a good effect, by almost instantaneously soothing the pain, which often accompanies inflammation. This mode of treatment I have found very successful in the scorbutic swelling of diseased gums, and it has removed affections which had previously resisted other remedies. When caries of the teeth is attended with pain, the application of monesia is sure to remove it in a few moments."

When all the ascertained facts are compared together, one is struck by the very peculiar tonic action of monesia on every organ. As its powers have been tried in more than four hundred cases, we may be allowed to consider monesia as a very useful remedy, under several circumstances, particularly scrofulous affections and uterine hæmorrhage. Hence to the art of healing it is a real acquisition; nor is it to be imagined that this tonic has any analogy with those already known,* quite lately a tannin ointment, and monesia ointment were tried and compared with each other, and the advantage was on the side of the latter. Moreover, it is clear that every medicine acts in its own way, and that there cannot be two whose special effects are the same. Well informed practitioners know that one purgative cannot be indifferently substituted for another; that every narcotic has not, in the same degree, the power of soothing and producing sleep; that the action of the various tonics is also very different; and that the general effects of medicines are like the difference of faces; many resemble each other at the first glance, but none can sustain an exact comparison.—*London, from Paris Medical Gazette.*

* There is some mistake in the original here, "que l'on ne croie pas que ce tonique ait quelque analogie avec ceux deja connus;" for, granting that its effects are not identical with those of any other onic, thereis a well marked anology.—*Translator.*

*Secondary Fistulæ of the Pleura in cases of Empyema and Pneumothorax.*—Dr. Stokes exhibited a drawing illustrative of this pathological condition, which had, he believed, been first described by Dr. Houghton in his article on Pneumothorax, in the Cylopædia of Practical Medicine. In the case then under consideration, the pleura presented the following appearances. The original fistula from within outwards, communicating with the cavity in the lung, was evident. But in addition to this opening, were several others, which had formed from without inwards; these presented oval patches, showing perforation of the serous membrane and subjacent structures, at the base of which were several circular openings, communicating with minute bronchial tubes, but not without any distinct tuberculous abscess. This condition seemed to require a very considerable duration of disease for its production; and Dr. Stokes was of opinion that these secondary fistulæ would account for some of the peculiarities in the physical signs of chronic empyema and pneumothorax.—*London Med. Gaz.*

*Ulceration of the Throat, extending to the lingual artery; death by hæmorrhage.*—Dr. Duncan presented the recent parts in this case. The patient, a young man, had been under treatment at the Adelaide Hospital, for ulcerated sore throat for some time, when he was suddenly attacked with hæmorrhage from the throat, which returned twice in the course of a fortnight. He had left the hospital, but was readmitted, and on the following day the bleeding returned with greater violence, and he was much exhausted. The ulceration was found to have attacked the right lingual artery, which presented a perforation capable of admitting a large sized probe. Dr. Duncan alluded to cases of the same kind which occurred under the care of the late Dr. M'Dowell, in one of which the external carotid had been tied with perfect success.—*Dublin Journal of Med. Science.*

*Softening of the Anterior Column of the Spinal Cord in its cervical portion.*—Dr. Power begged to draw the attention of the Society to a well marked and recent specimen of acute softening of the anterior column of the spinal cord. The patient, a woman, aged 50, was suddenly attacked with paralysis of motion in the upper and lower extremities. The bladder and rectum were unaffected; a slight power of motion remained in the limbs. There was no loss of sensation; no fever, headach, or disturbance of intellect. Sensation in the paralysed portions was perfect. Soon afterwards she was attacked with dyspnœa, and her breathing became diaphragmatic: ultimately the diaphragm became paralysed, and death took place with great dyspnœa. The spinal column was opened on the following day, and the cervical portion of the medulla spinalis was found softened to a great degree.—*Ibid.*

